# Analgesia and unwanted benzodiazepine effects in point-mutated mice expressing only one benzodiazepine-sensitive GABA_A_ receptor subtype

**DOI:** 10.1038/ncomms7803

**Published:** 2015-04-13

**Authors:** William T. Ralvenius, Dietmar Benke, Mario A. Acuña, Uwe Rudolph, Hanns Ulrich Zeilhofer

**Affiliations:** 1 Institute of Pharmacology and Toxicology, University of Zurich, Winterthurerstrasse 190, CH-8057 Zurich, Switzerland; 2 Center for Neuroscience Zurich (ZNZ), Winterthurerstrasse 190, CH-8057 Zurich, Switzerland; 3 Laboratory of Genetic Neuropharmacology, McLean Hospital, 115 Mill Street, Belmont, Massachusetts 02478, USA; 4 Department of Psychiatry, Harvard Medical School, 401 Park Drive, Boston, Massachusetts 02215, USA; 5 Institute of Pharmaceutical Sciences, Swiss Federal Institute of Technology (ETH) Zurich, Vladimir-Prelog-Weg 4, CH-8093 Zurich, Switzerland

## Abstract

Agonists at the benzodiazepine-binding site of GABA_A_ receptors (BDZs) enhance synaptic inhibition through four subtypes (α1, α2, α3 and α5) of GABA_A_ receptors (GABA_A_R). When applied to the spinal cord, they alleviate pathological pain; however, insufficient efficacy after systemic administration and undesired effects preclude their use in routine pain therapy. Previous work suggested that subtype-selective drugs might allow separating desired antihyperalgesia from unwanted effects, but the lack of selective agents has hitherto prevented systematic analyses. Here we use four lines of triple GABA_A_R point-mutated mice, which express only one benzodiazepine-sensitive GABA_A_R subtype at a time, to show that targeting only α2GABA_A_Rs achieves strong antihyperalgesia and reduced side effects (that is, no sedation, motor impairment and tolerance development). Additional pharmacokinetic and pharmacodynamic analyses in these mice explain why clinically relevant antihyperalgesia cannot be achieved with nonselective BDZs. These findings should foster the development of innovative subtype-selective BDZs for novel indications such as chronic pain.

## Abstract (web summary)

Benzodiazepines (BDZs) target GABA_A_ receptors to alleviate pain but these also cause side effects. Here the authors use mice in which only one GABA_A_ receptor is BDZ-sensitive at a time to identify α2GABA_A_ as the receptor that provides maximal analgesic activity but minimal side-effects in response to BDZs.

## Graphical abstract

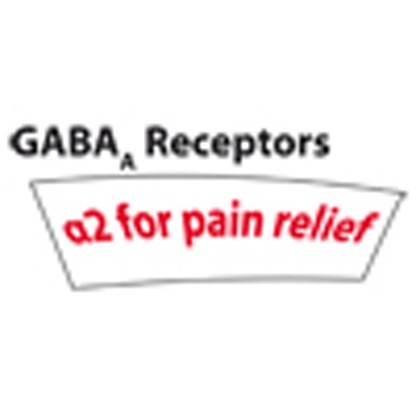

Chronic pain is a severe medical condition affecting hundreds of millions of patients worldwide. It is widely accepted that diminished inhibition in pain-processing circuits of the spinal dorsal horn is a major contributor to different chronic pain forms^1^,^2^,^3^,^4^. We have previously demonstrated that local spinal application of BDZ site ligands that positively modulate GABA_A_R function alleviates neuropathic and inflammatory pain in rodents^5^. Translation of these results to routine systemic pain treatment, however, requires a separation of desired antihyperalgesia from unwanted side effects. This separation appears potentially feasible based on the existence of different GABA_A_R subtypes.

Most GABA_A_Rs in the brain and spinal cord are heteropentameric ion channels composed of two α, two β and one γ2 subunit^6^. The high-affinity binding site for BDZs is formed by an interface between one α subunit and the γ2 subunit. High-affinity binding of BDZs at this site requires the presence of a histidine residue at a conserved site in the N-terminal domain of the α subunit. This conserved histidine is present in α1, α2, α3 or α5 subunits, but not in the α4 and α6 subunits^7^. Mutation of the histidine residue into an arginine dramatically reduces the affinity of GABA_A_Rs to BDZs without changing their responses to GABA^8^. The generation of histidine to arginine (H→R) point-mutated mice for each of the four BDZ-sensitive GABA_A_R subunits has allowed attributing the different *in vivo* effects of BDZs to defined GABA_A_R subtypes^9^. Most importantly, it was shown that the sedative effects of BDZs were strongly reduced in mice carrying the H→R point mutation in their α1 subunits^10^,^11^, while point-mutating the α2 subunits led to a loss in the anxiolytic effects of BDZs^12^. Using this approach, we could demonstrate that mice whose α2GABA_A_Rs had been rendered BDZ-insensitive show drastically reduced antihyperalgesic responses to spinal diazepam (DZP)^5^. A general consensus on the question, which GABA_A_R subtype should best be targeted to achieve maximal antihyperalgesic responses and to best avoid undesired effects has not been reached. A similarly open question is why classical BDZs are largely devoid of analgesic actions in patients.

Highly selective tool compounds that would allow addressing these questions pharmacologically are still lacking^13^. For this reason, we have generated triple GABA_A_R point-mutated mice, in which only a single GABA_A_R subtype remains BDZ-sensitive. We designate these mice hereafter as HRRR, short for α1H;α2R;α3R;α5R (that is, a mouse in which only α1GABA_A_Rs are left BDZ-sensitive) and, accordingly, as RHRR, RRHR, RRRH for mouse lines, in which either only α2, α3 or α5 subunits remain BDZ-sensitive. In these mice, the action of the classical normally nonselective BDZ agonist DZP should mimic the action of fully subtype-selective compounds. Such a genetic approach also avoids possible confounding factors of subtype-selective compounds such as pharmacokinetic differences and unknown specificity profiles of drug metabolites. With the use of triple GABA_A_R point-mutated mice, we were not only able to perform a systematic analysis on the GABA_A_R subtypes best targeted for optimal antihyperalgesia and least-pronounced side effects; however, we also provide an explanation why classical (nonselective) BDZs are largely devoid of clinically relevant analgesic properties.

## Results

### Spinal distribution of subtype-specific BDZ-binding sites

**Figure 1:**
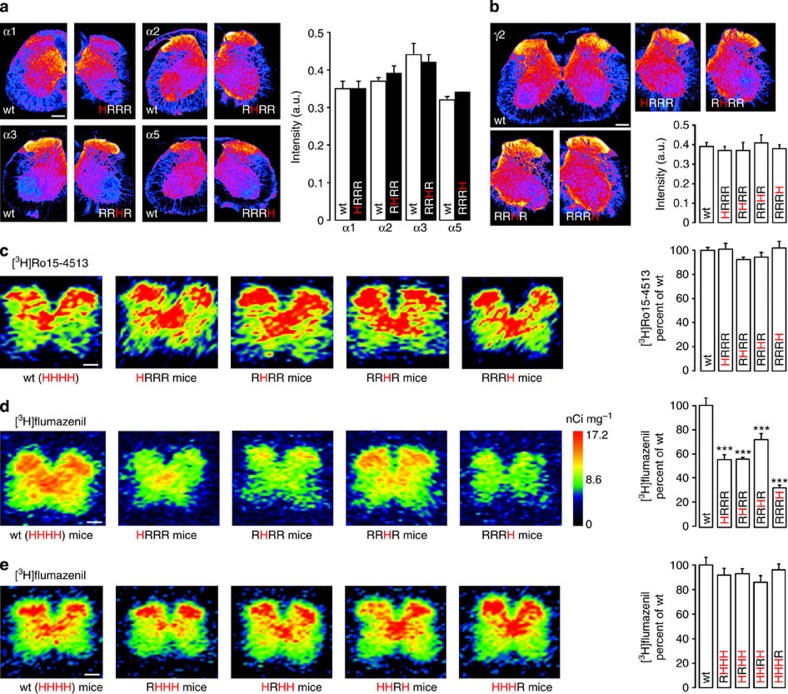
Distribution of the four subtypes of BDZ-sensitive GABA_A_Rs in the spinal cord. (**a**,**b**) Immunohistochemistry. (**a**) Localization of GABA_A_R α subunits in spinal cords of wt mice and of the four types of GABA_A_R triple point-mutated mice. Pseudocolour images display receptor density (lowest to highest density, blue to yellow). Right: quantitative comparison of expression levels in wt and triple point-mutated mice. Two-way analysis of variance (ANOVA) for interaction (α subunit × genotype): F(7,42)=0.53, *P*=0.68, *n*=6 mice (three sections each) for all groups, except RRHR, where *n*=7 mice. Panel **b** same as **a**, but for γ2 subunits. ANOVA followed by Dunnett's *post hoc* test. F(4,15)=0.32; *P*⩾0.94. *n*=4 mice (three sections each) for all genotypes. (**c**,**d**) Autoradiography. (**c**) [^3^H]Ro15-4513-binding sites indicate the distribution of all α/γ GABA_A_Rs independent of their BDZ sensitivity in wt mice and the four types of GABA_A_R triple point-mutated mice. H and R indicate homozygous wt and point-mutated alleles (for α1/α2/α3/α5 subunits), respectively. Right: quantification of [^3^H]Ro15-4513-binding sites in the dorsal horn. ANOVA followed by Dunnett's *post hoc* test with wt as control. F(4,10)=2.89, *P*⩾0.18, *n*=3, for all genotypes (>30 sections each). (**d**) Same as **c**, but distribution of BDZ-sensitive GABA_A_Rs labelled with [^3^H]flumazenil. ANOVA followed by Dunnett's *post hoc* test with wt as control. F(4,55)=40.1, ***P<0.001, *n*=11, 12, 12, 12 and 14 mice (>10 sections each), for wt (HHHH), HRRR, RHRR, RRHR and RRRH mice, respectively. (**e**) Same as (**d**), but wt and GABA_A_R single point-mutated mice. F(4,42)=0.441, *P*>0.66, *n*=11, 9, 9, 9 and 9 mice, for wt(HHHH), RHHH, HRHH, HHRH and HHHR mice, respectively. All scale bars, 200 μm. All quantitative data mean±s.e.m.

In the first series of experiments, we characterized the distribution pattern of the four subtypes of BDZ-sensitive GABA_A_Rs (‘BDZ receptors') in the spinal dorsal horn of triple point-mutated mice. Using immunocytochemistry, we verified that neither the regional distribution nor the expression levels of α1, α2, α3 and α5 subunits and of the γ2 subunit differed between wild-type (wt) mice and the four strains of triple point-mutated mice (Fig. 1a,b). Similarly, the total number of BDZ-binding sites (wt receptors plus H→R point-mutated receptors) quantified through [^3^H]Ro15-4513 autoradiography was unchanged (Fig. 1c). Spinal autoradiography with [^3^H]flumazenil that binds with high affinity only to nonpoint-mutated GABA_A_Rs allowed a quantitative analysis of each of the four subtypes of BDZ-binding sites in isolation. In wt mice, [^3^H]flumazenil exhibited specific binding throughout the spinal grey matter with enrichment in the dorsal horn and around the central canal. The density of binding sites in the dorsal horn was highest for the α3GABA_A_R subtype, followed by α2 and α1, and lowest for the α5 subtype (Fig. 1d). The α1 subtype was concentrated around the central canal, α2 was most abundant in the superficial dorsal horn, where nociceptive fibres terminate, and α3 was found throughout the dorsal horn and around the central canal, while α5 was generally weak. The distribution of subtype-specific [^3^H]flumazenil binding in the four strains of GABA_A_R triple point-mutated mice matched the distribution of the subunits determined with immunocytochemistry on a gross scale. However, the sum of the binding sites in the four strains of triple point-mutated mice was about twice as high as the total number of binding sites in wt mice. The results from the immunocytochemistry and [^3^H]Ro15-4513 autoradiography largely rule out changes in protein expression as the underlying cause. Instead, the [^3^H]flumazenil data are consistent with a high prevalence of GABA_A_Rs containing two different α subunits^14^,^15^ and with a model of the GABA_A_R assembly in which nonpoint-mutated α subunits have a higher probability for interaction with the γ2 subunit than H→R point-mutated subunits^16^. This conclusion is further supported by the only marginal reduction of BDZ binding observed in spinal cords of the four single point-mutated mouse lines (Fig. 1e).

### Antihyperalgesia by single BDZ-sensitive GABA_A_Rs subtypes

**Figure 2:**
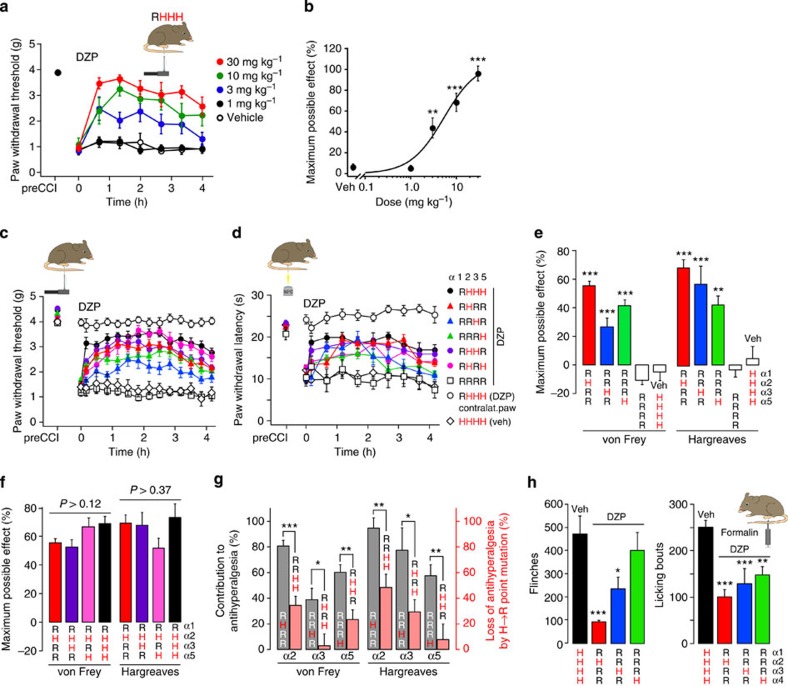
Antihyperalgesia by per oral DZP in GABA_A_R point-mutated mice. (**a**) Reversal of mechanical hyperalgesia by DZP in α1 point-mutated (RHHH) mice assessed with von Frey filaments 7 days after CCI surgery. (**b**) Dose–response relationship. Data were fitted to Hill's equation. ***P<0.001; **P<0.01 versus vehicle (ANOVA F(4,28)=29.0 followed by Dunnett's *post hoc* test, *n*=7, 7, 6, 7 and 6 mice, for vehicle and 1, 10 and 30 mg kg^−1^ DZP, respectively). (**c,d**) Reversal of mechanical (**c**) and heat hyperalgesia (**d**) by DZP (10 mg kg^−1^) 1 week after the CCI surgery. All mice except wt carried the H→R point mutation in α1GABA_A_Rs to avoid sedation. (**e**) Comparison of antihyperalgesia (determined from 70 to 130 min after DZP administration) in GABA_A_R triple point-mutated mice. ***P<0.001; **P<0.01; versus vehicle-treated wt mice (ANOVA followed by Dunnett's *post hoc* test, F(4,54)=36.2 and F(4,53)=15.1 for von Frey and Hargreaves tests, respectively). No antihyperalgesia by DZP occurred in quadruple GABA_A_R point-mutated mice (*P*=0.85 and 0.94). (**f**) Comparison of antihyperalgesia by DZP in RHRR mice with that of mice carrying, in addition, BDZ-sensitive α3 and/or α5GABA_A_Rs. No significant differences were observed (ANOVA followed by Dunnett's *post hoc* test, von Frey: F(3,57)=2.96; *P*⩾0.12. Hargreaves: F(3,44)=0.98; *P*⩾0.37). (**g**) Antihyperalgesia in triple GABA_A_R point-mutated mice with only one DZP-sensitive GABA_A_R subtype (grey columns) compared with antihyperalgesia lost through H→R point mutation of the same subunit (light red columns). Antihyperalgesia in triple point-mutated mice is expressed as percentage of antihyperalgesia in RHHH mice. Loss of antihyperalgesia was calculated as the reduction in antihyperalgesia relative to RHHH mice. ***P<0.001; **P<0.01; *P<0.05 (unpaired *t*-tests). Number of mice in **c**–**g**: vehicle-treated wt (von Frey/Hargreaves test) *n*=7/11, RHHH *n*=15/12, RHRR *n*=18/19, RRHR *n*=13/9, RRRH *n*=11/12, RHHR *n*=19/10, RHRH *n*=9/7, RRRR *n*=9/6, RRHH *n*=8/19. (**h**) Formalin test. Reduction by DZP (10 mg kg^−1^) in the number of flinches and licking bouts compared with vehicle-treated mice. ***P<0.001; **P<0.01; *P<0.05 (ANOVA followed by Dunnett's *post hoc* test. Flinches, F(3,24)=6.71; licking bouts, F(4,25)=10.7. *n*=9, 6, 7 and 6 mice, for vehicle-treated wt and DZP-treated RHRR, RRHR and RRRH mice, respectively. All data points are mean±s.e.m.

We next used the triple point-mutated mice to predict pharmacological actions of subtype-selective BDZ site agonists and treated them with DZP, a classical nonselective BDZ, whose agonistic activity was restricted to a single GABA_A_R subtype in triple point-mutated mice. We first focused on the antihyperalgesic potential of such an approach (that is, the potential to reduce neuropathy-induced hyperalgesia) and tested DZP in mice subjected to the chronic constriction injury (CCI; ref. 17) model. Baseline mechanical and heat sensitivity were statistically indistinguishable in all strains of mice analysed and all strains of mice developed similar hyperalgesia after CCI (Table 1). All subsequent experiments with DZP were performed on an α1R point-mutated background to avoid DZP-induced sedation, which is a potential confounding factor in behavioural pain tests. We first tested different doses of systemic (per os (p.o.)) DZP in α1 point-mutated RHHH mice (Fig. 2a,b). On the basis of these results, we chose a dose of 10 mg kg^−1^ body weight, which is close to the ED_70_, for subsequent experiments. We then asked which GABA_A_R subtype exerts the strongest antihyperalgesic action and tested the effects of DZP on mechanical (von Frey filament test) and heat (Hargreaves test) hyperalgesia in the four strains of GABA_A_R triple point-mutated mice (Fig. 2c–e). For both stimuli, the strongest effect was achieved in RHRR mice in which only α2GABA_A_Rs were BDZ-sensitive. Exclusive targeting of α3 and α5GABA_A_Rs (in RRHR and RRRH mice) also elicited antihyperalgesia but to a smaller extent. Importantly, mice in which all four BDZ receptors had been point-mutated (RRRR mice) did not show any antihyperalgesic response. We next asked whether targeting of a second or third GABA_A_R subtype in addition to α2 would increase antihyperalgesic efficacy and compared antihyperalgesic responses in triple point-mutated mice with those in mice carrying only one or two point-mutated receptors. We found only small and statistically insignificant differences indicating that adding activity at a subtype different from α2 did not significantly increase antihyperalgesia (Fig. 2f).

The autoradiography data shown in Fig. 1d suggest a high prevalence of GABA_A_Rs with two different α subunits (‘mixed GABA_A_Rs') in the spinal cord. Because the sum of the four receptor subtypes detected in the triple point-mutated mice greatly exceeded the total number of [^3^H]flumazenil binding sites in the wt mice, the pharmacological effect of a given GABA_A_R subtype may be overestimated when tested in triple point-mutated mice. To address this issue, we compared the level of antihyperalgesia achieved in the triple point-mutated mice with the loss in antihyperalgesia that occurs after mutation of the same subunit (Fig. 2g). For both types of sensory tests (Hargreaves and von Frey filament) and for all three α subunits under study, we found that the level of antihyperalgesia achieved in the triple point-mutated mice always exceeded the loss in antihyperalgesia by mutation of the same subunit consistent with the high prevalence of mixed GABA_A_Rs in the spinal cord. It is important to note that the rank order of antihyperalgesic efficacies was the same in experiments with triple point-mutated mice and in loss-of-function experiments in single and double point-mutated mice, and also identical to those found in a pain model employing chemical nociceptor activation instead of neuropathy (Fig. 2h).

**Figure 3:**
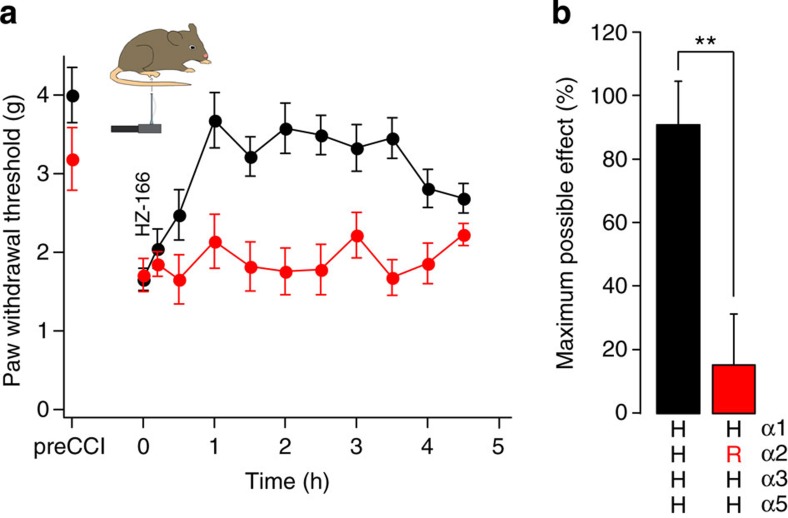
Antihyperalgesia by the non-sedative BDZ HZ-166 occurs through α2GABA_A_Rs. (**a**) HZ-166 (16 mg kg^−1^, i.p.,) almost completely reversed CCI-induced mechanical sensitization in wt mice (HHHH, black), but not in α2 (H101R) mice (HRHH mice, red). (**b**) Statistical analysis. **P<0.01 (unpaired *t*-test), *n*=7 and 8 mice, for wt and α2 (H101R) mice. All data points are mean±s.e.m.

Taken together, these results support a major contribution of α2GABA_A_Rs to antihyperalgesia in different pain models. However, because all experiments had to be conducted on an α1 point-mutated background to avoid confounding sedation, the relevance of α2GABA_A_Rs might be overestimated if ‘pain-relevant' α2GABA_A_Rs contained also α1 subunits. To address this potential caveat we used HZ-166 (ref. 18), a novel benzodiazepine site ligand with an improved α2/α1 selectivity ratio^19^ and significant analgesic activity already at non-sedative doses^20^ (Fig. 3). Antihyperalgesia by HZ-166 was almost completely blocked in single α2(H101R) point-mutated (HRHH) mice, further confirming that the ‘pain-relevant' GABA_A_Rs exhibit an α2 pharmacology.

### Potential undesired BDZ effects in triple point-mutated mice

**Figure 4:**
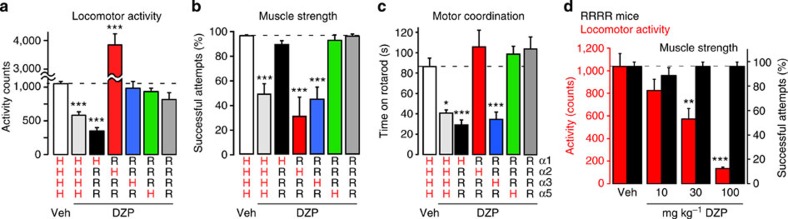
Sedation, muscle relaxation and motor coordination in GABA_A_R-mutated mice. Effects of DZP (10 mg kg^−1^, p.o.) on locomotor activity in the open field test (**a**), on muscle relaxation in the horizontal wire test (**b**), and on motor coordination in the rotarod test (**c**). ***P<0.001, *P<0.05 significant versus vehicle-treated wt (HHHH) mice (ANOVA followed by Dunnett's *post hoc* test). Statistics: locomotor activity F(6,177)=79.5 (*n*=106, 26, 8, 8, 9, 13 and 13 mice, for vehicle and DZP-treated wt mice, and DZP-treated HRRR, RHRR, RRHR, RRRH and RRRR mice, respectively). Horizontal wire F(6,165)=44.0 (*n*=109, 14, 8, 8, 9, 13 and 10 mice). Rotarod F(6,41)=11.5 (*n*=8, 5, 6, 7, 6, 8 and 8 mice). (**d**) Effects of DZP on locomotor activity and horizontal wire performance in quadruple GABA_A_R point-mutated (RRRR) mice. ***P<0.001; **P<0.05 significant versus vehicle (ANOVA followed by Dunnett's *post hoc* test) F(3,33)=13.4 (locomotor activity), *n*=9, 13, 8 and 7 mice, for vehicle, and 10, 30 and 100 mg kg^−1^ DZP; F(3,30)=0.44; *P*>0.60 (horizontal wire test), *n*=9, 10, 8 and 7 mice, for vehicle, and 10, 30 and 100 mg kg^−1^ DZP. All data points are mean±s.e.m.

We next used the same triple point-mutated mouse approach to investigate non-pain-related effects. In these experiments, we focused on diminished locomotor activity (as a surrogate parameter of sedation), muscle strength and motor coordination. No significant differences were observed in the behaviour of drug-naive wt and point-mutated mice (Table 2). The DZP treatment strongly reduced locomotor activity in wt mice and in mice with BDZ-sensitive GABA_A_Rs of only the α1 subtype (HRRR mice; Fig. 4a). No sedative effects were observed in any of the other triple point-mutated mice. Mice with only α2 BDZ-sensitive GABA_A_Rs (RHRR mice) even showed a strong increase in locomotor activity, which may originate from the anxiolytic effect of DZP occurring through α2GABA_A_Rs (ref. 12). Impairment of muscle strength was assessed in the horizontal wire test. Significant muscle relaxation was detected in wt mice and in mice with BDZ-sensitive GABA_A_Rs of either only the α2 (RHRR mice) or α3 subtype (RRHR mice; Fig. 4b). Motor coordination, tested in the rotarod test, was significantly impaired by DZP in mice with only α1 (HRRR mice) and with only α3 BDZ-sensitive GABA_A_Rs (RRHR mice; Fig. 4c). Quadruple point-mutated (RRRR) mice were completely protected from DZP-induced muscle relaxation and motor impairment. They did, however, show a trend towards reduced locomotor activity (compare Fig. 4a). We therefore assessed changes in locomotion also after higher DZP doses in the quadruple point-mutated mice and found significant and dose-dependent impairment starting at 30 mg kg^−1^ (Fig. 4d). Unlike α1GABA_A_R-mediated sedation, impairment of muscle strength was absent in the quadruple point-mutated mice even at doses ⩾30 mg kg^−1^. The sedative action of DZP remaining in the quadruple point-mutated mice may be attributed to a low-affinity BDZ-binding site at α1GABA_A_Rs described earlier^21^,^22^.

Our findings on the contribution of α1, α2 and α3GABA_A_Rs to sedation, anxiolysis and muscle relaxation confirm previous studies using single GABA_A_R point-mutated mice^11^,^12^,^23^. The results from our experiments with triple point-mutated mice on motor coordination, however, differ from those obtained with single point-mutated mice^11^,^12^. This discrepancy may arise from the involvement of mixed GABA_A_Rs containing α1 and α3 subunits. We therefore tested whether motor coordination would also be impaired by TP003, an α3GABA_A_R-selective BDZ site agonist^24^. Two hours after treatment with TP003 (10 mg kg^−1^, p.o.), the time to fall off the rod was similarly decreased in wt and RRHR mice (by 43.9±10.2%, paired *t*-test *P*<0.05, *n*=5, in wt mice, and by 38.5±6.4, *n*=5, *P*<0.05), while HHRH and vehicle-treated wt mice showed no impairment (0.7±12.0%, *n*=5, *P*=0.91 and −4.7±9.2%, *n*=5, *P*=0.59).

### Liability to tolerance development

**Figure 5:**
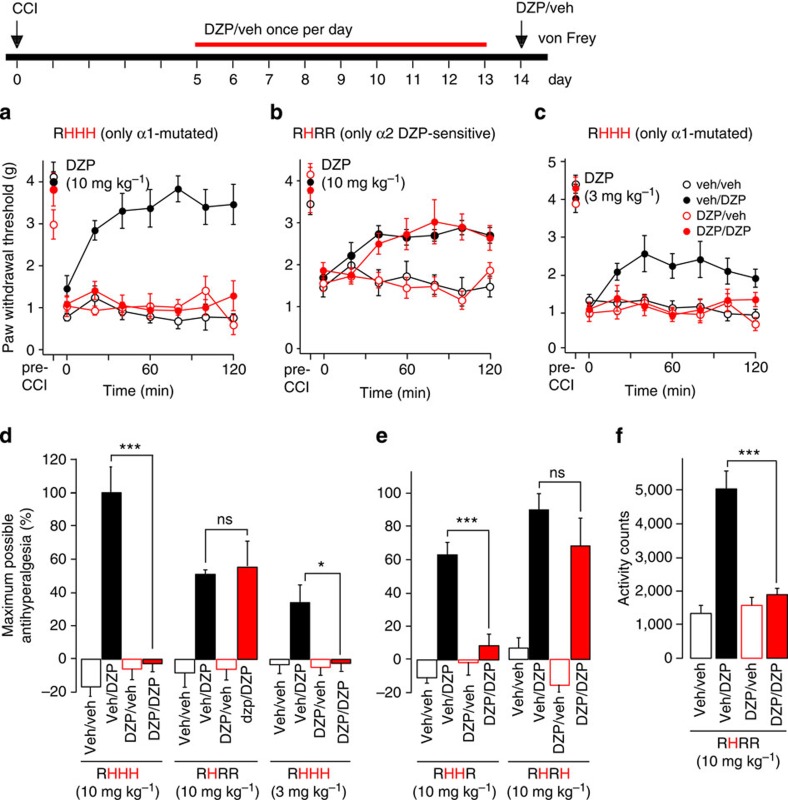
Tolerance liability against the antihyperalgesic effects of DZP. Starting on day 5 after the CCI surgery, mice were treated with DZP or vehicle once daily for nine consecutive days. On day 10, mice were given either DZP or vehicle, and antihyperalgesic effects were measured for 2 h. (**a**) Complete loss of antihyperalgesic activity was observed after 9-day DZP treatment (10 mg kg^−1^, p.o.) in RHHH mice. (**b**) Same as **a**, but RHRR triple point-mutated mice. These mice were completely protected from tolerance development against DZP-induced antihyperalgesia. (**c**) Same as **a**, but lower dose of DZP (3 mg kg^−1^, p.o.). Tolerance in RHHH mice still developed at a lower dose of DZP, which induced an antihyperalgesic effect similar to that of 10 mg kg^−1^ in RHRR mice. (**d**) Statistical comparison. Two-way ANOVA for the interaction pretreatment × acute treatment (F(3,25)=32.5 (*n*=5, 8, 7 and 8 mice for veh/veh, veh/DZP, DZP/veh and DZP/DZP groups, respectively); F(3,25)=0.014 (*n*=7, 8, 7 and 6); F(3,24)=5.97 (*n*=8, 7, 7 and 6), for data shown in **a**–**c**). Maximum antihyperalgesic activity was calculated for the interval between 80 and 120 min after DZP administration. (**e**) Double GABA_A_R point-mutated mice (RHHR and RHRH), in which α3GABA_A_R or α5GABA_A_R were left BDZ-sensitive in addition to α2GABA_A_R. Tolerance development required the additional presence of DZP-sensitive α3GABA_A_R. Two-way ANOVA for the interaction pretreatment × acute treatment. RHHR mice: F(3,22)=22.1 (*n*=6, 6, 7 and 6 mice for veh/veh, veh/DZP, DZP/veh and DZP/DZP groups, respectively); RHRH mice: F(3,21)=0 (*n*=6 for all groups). (**f**) Unlike antihyperalgesic effects, anxiolytic effects of DZP were still susceptible to tolerance development in RHRR mice after 9-day treatment with DZP (10 mg kg^−1^, p.o.). Two-way ANOVA F(3,26)=28.3 (*n*=8, 7, 7 and 7) for the interaction pretreatment × acute treatment. ***P=0.001; *P=0.05. All data points are mean±s.e.m.

Another major limitation of classical BDZs is their liability to tolerance development (that is, the loss of activity during prolonged use). We tested whether this tolerance would also occur for the antihyperalgesic actions of BDZs and whether it could be prevented by selectively targeting α2GABA_A_Rs. To this end, we applied again CCI surgery and treated mice of the different strains for nine consecutive days with 10 mg kg^−1^ DZP or vehicle p.o. once daily. On the 10th day, both groups of mice were split into two subgroups, treated with either 10 mg kg^−1^ DZP or vehicle, and mechanical hypersensitivity was monitored for 2 h after drug application. We first did this experiment in single α1 point-mutated (RHHH) mice to avoid confounding sedation (Fig. 5a,d). While DZP-naive mice responded with an almost complete reversal of hyperalgesia, mice chronically pretreated with DZP showed no antihyperalgesic response. We then performed this experiment in triple point-mutated RHRR mice with only α2 BDZ-sensitive GABA_A_Rs and found that these mice were completely protected from tolerance development (Fig. 5b,d). In the latter experiment, the antihyperalgesic response was generally lower than in α1 point-mutated (RHHH) mice. We therefore repeated the first experiment with a lower dose of DZP (3 mg kg^−1^) to exclude the possibility that the absence of tolerance in the RHRR mice was due to a smaller DZP effect. With this lower dose, complete tolerance still developed in RHHH mice (Fig. 5c,d). These experiments indicate that activity at α3 or α5GABA_A_Rs was necessary to induce antihyperalgesic tolerance. We therefore tested double point-mutated mice in which α3GABA_A_Rs or α5GABA_A_Rs were left BDZ-sensitive in addition to α2GABA_A_Rs (RHHR and RHRH mice). Complete tolerance developed in RHHR mice, but not in RHRH mice, indicating that activity at α3GABA_A_Rs was necessary for induction of antihyperalgesic tolerance in mutated mice (Fig. 5e). In this set of experiments, we finally tested whether the same subtype dependence would also be found for tolerance against α2GABA_A_R-mediated anxiolysis (Fig. 5f). Unlike antihyperalgesic tolerance, anxiolytic tolerance (measured as increased locomotor activity in the open field) still developed in RHRR mice. This dissociation suggests that tolerance development against antihyperalgesia is not a receptor or cell-autonomous process^25^ but rather occurs at the circuit level.

### Why classical BDZs lack clinically relevant analgesic properties

**Figure 6:**
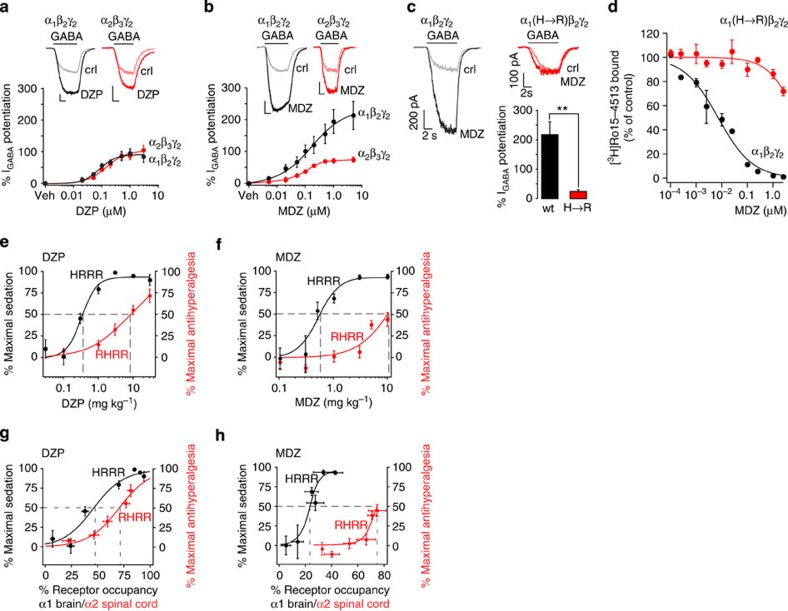
Activity of DZP and MDZ at α1 and α2GABA_A_Rs, and PK/PD modelling. (**a**,**b**) Potentiation of recombinant α1β2γ2 and α2β3γ2 GABA_A_R currents by 1 μM DZP (**a**) and 1 μM MDZ (**b**). GABA concentration was EC_5_ (5 μM for α1β2γ2 and 2 μM for α2β3γ2). *n*=6 for both drugs and all concentrations. Scales bars, 200 pA, 2 s. (**c**,**d**) The H101R mutation prevented potentiation of α1β2γ2 GABA_A_R currents by MDZ (**P<0.01, *n*=5, unpaired *t*-test; **c**) and binding of MDZ to α1β2γ2 GABA_A_Rs (*n*=6 for all concentrations in wt and mutated receptors; **d**). (**e**–**h**) α1GABA_A_R-mediated sedation and α2GABA_A_R-mediated antihyperalgesia by DZP and MDZ. Sedation (black symbols) in HRRR mice was assessed in the open field test and expressed as percent maximum possible effect determined from the reduction in activity compared with vehicle-treated mice of the same genotype. Antihyperalgesia (red symbols) in RHRR mice was determined as the increase in mechanical withdrawal thresholds compared with pre-drug values 1 week after the CCI surgery. (**e**,**f**) Dose–response relationships. Data were fitted to Hill's equation with baseline fixed at 0. Sedation and antihyperalgesia were determined between 15 and 90 min after oral DZP (**e**) or between 15 and 60 min after oral MDZ (**f**). Mice were killed immediately afterwards (that is, at the time point of maximal effects), and brains and spinal cords were removed for further analyses. (**g**,**h**) Dependence on receptor occupancy (RO). (**g**) Sedation versus RO in brains of HRRR mice treated with DZP (0.03, 0.1, 0.3, 1, 3, 10 and 30 mg kg^−1^, *n*=6 mice for all doses). Antihyperalgesia versus RO in lumbar spinal cords of RHRR mice treated with 0.1, 1, 3, 10 or 30 mg kg^−1^ DZP, *n*=6 mice for all doses. (**h**) Same as **g** but MDZ. Sedation 0.1 (*n*=6 mice), 0.3 (7), 0.5 (7), 1.0 (7), 3 (6) and 10 (4) mg kg^−1^. Antihyperalgesia 0.1 (*n*=3), 0.3 (5), 1.0 (9), 3 (8), 5 (7) and 10 (7) mg kg^−1^ MDZ. Data were fitted to sigmoidal functions. Data shown in **e**,**g** (on DZP) and in **f**,**h** (on MDZ) are from the same two groups of mice. All data points are mean±s.e.m.

Our present results and previous pain studies employing subtype-selective BDZ site agonists in rodents^5^,^20^,^26^,^27^,^28^,^29^ contrast with the lack of a clear analgesic or antihyperalgesic action of classical BDZs in human patients^30^,^31^. Apart from species differences and differences between disease models and actual disease in human patients, we found one possible explanation particularly worth studying. The doses and the degrees of receptor activation required for a relevant effect might be significantly higher in case of antihyperalgesia than of sedation. As a consequence, antihyperalgesia would occur in patients only at doses already inducing strong sedation. The availability of the triple point-mutated mice allowed us to directly compare the doses and levels of receptor occupancy at the relevant GABA_A_R subtypes and sites. For these experiments, we chose midazolam (MDZ) as a second classical BDZ in addition to DZP. First, we determined for both drugs their α2/α1 selectivity profiles in electrophysiological experiments on recombinant GABA_A_Rs. As expected, we found that DZP potentiated α1/β2/γ2 and α2/β3/γ2 GABA_A_Rs with similar efficacy and potency (Fig. 6a and Table 3). By contrast, MDZ potentiated α1/β2/γ2 GABA_A_Rs more than twice as much as α2/β3/γ2 GABA_A_Rs (Fig. 6b and Table 3). We then verified that the H→R point mutation blocked not only DZP effects but also MDZ binding and GABA_A_R potentiation (Figs 6c,d; see also ref. 32). Next, we compared the dose dependency of DZP- and MDZ-induced antihyperalgesia in mice with only α2 BDZ-sensitive GABA_A_Rs (RHRR mice) with that of DZP- and MDZ-induced sedation in mice with only α1 BDZ-sensitive GABA_A_Rs (HRRR mice). We found that half maximal sedation occurred already at a doses of 0.33±0.05 and 0.52±0.11 mg kg^−1^ (mean±s.d.) for DZP and MDZ, respectively, while half maximal antihyperalgesia required 8±6 mg kg^−1^ (DZP) and 10.4±2.0 mg kg^−1^ (MDZ). A rightward shift of the response curve was also observed when the degrees of receptor occupancy required for antihyperalgesia and for sedation were compared. Half maximal sedation was reached when DZP had bound 47±6% brain α1GABA_A_Rs, while half maximal antihyperalgesia required 71±2% occupancy at spinal α2GABA_A_Rs. In case of MDZ, the required receptor occupancies were even further apart (24±2% of brain α1GABA_A_Rs and 71±2% of spinal α2GABA_A_Rs) consistent with the even less favourable α2/α1 selectivity ratio of MDZ. These data indicate that, when applied to wt mice, the DZP and MDZ doses needed for half maximal antihyperalgesia induce an almost complete (∼95%) reduction in locomotor activity, while at non-sedative doses both BDZs would not induce significant antihyperalgesia. Dose-limiting sedation is therefore the most likely reason for the absence of a relevant antihyperalgesic activity of classical, nonselective BDZs in human patients.

## Discussion

In the present study we have employed triple GABA_A_R point-mutated mice to characterize the pharmacological actions expected from yet-to-be-developed subtype-selective BDZ-binding site agonists. Our study can be viewed as a ‘restriction-of-function' approach (‘what effect of DZP remains when only a single GABA_A_R subtype is targeted?') as opposed to previous loss-of-function studies in single GABA_A_R point-mutated mice (‘which effects of DZP are lost or reduced when activity at one GABA_A_R subtype is abolished compared with DZP-treated wt mice?'). With respect to sedation, anxiolysis and the muscle-relaxant action, the present study confirms previous results obtained with single point-mutated mice: sedative actions of BDZs occur through α1GABA_A_R (refs 10, 11), anxiolytic effects through α2GABA_A_Rs (ref. 12) and muscle relaxation through α2 and α3GABA_A_R (ref. 23).

Discrepancies between results from restriction-of-function and loss-of-function approaches were observed for impairment of motor coordination. The present study shows that undesired impairment of motor coordination is caused by DZP in HRRR and RRHR mice (that is, it is evoked when either α1 or α3GABA_A_Rs are specifically targeted), while previous reports in the respective single point-mutated mice failed to provide evidence for an involvement of these GABA_A_R subtypes^11^,^12^. This discrepancy is consistent with the idea that DZP impairs motor coordination through mixed GABA_A_Rs containing an α1 and an α3 subunit (α1/α3GABA_A_Rs). Immunohistochemical analyses have shown that co-expression of α1 and α3 GABA_A_R subunits occurs in subsets of central neurons^33^,^34^, and biochemical data have provided direct evidence for the presence of different α subunits within the same GABA_A_R complex^15^. In particular, α3 containing GABA_A_Rs occur mainly as mixed α1/α3GABA_A_Rs (refs 14, 15). Furthermore, biochemical data also suggest that in mixed GABA_A_Rs with one point-mutated α subunit the nonpoint-mutated (wt) subunit has a higher probability for interaction with the γ2 subunit than the point-mutated α subunit^15^,^16^. Results from loss-of-function analysis in single point-mutated mice and from restriction-of-function analyses in triple point-mutated mice should therefore be seen as lower and upper limits for the contribution of a given GABA_A_R subtype to an *in vivo* drug action. Conversely, the lack of sedation in α1 point-mutated mice^10^,^11^ and of anxiolysis in α2 point-mutated mice^12^ suggests that these actions occur through GABA_A_Rs with only a single type of α subunit. In line with this conclusion is that the majority of GABA_A_Rs in the brain are pure α1GABA_A_Rs (α1/α1GABA_A_Rs)^15^. Similarly, BDZ-mediated anxiolysis occurs through pure α2GABA_A_Rs, with α2/α2GABA_A_Rs being the single most prevalent isoform of α2 containing GABA_A_Rs at supraspinal sites^15^.

As demonstrated by our autoradiography experiments (compare Fig. 1d,e), mixed GABA_A_Rs make up the vast majority of GABA_A_Rs in the spinal cord. Following the model described above, one would expect that in case of mixed GABA_A_Rs the single point mutation approach would yield smaller effects than the triple point mutation strategy. For instance, in case of an αx/αyGABA_A_R neither the αx single point mutation nor the αy single point mutation would abolish BDZ sensitivity; however, both triple point-mutated mice with only αx or only αy remaining BDZ-sensitive would yield full behavioural effects. Results for antihyperalgesia obtained in the present study were consistent with a contribution of mixed GABA_A_Rs to antihyperalgesia. For all three contributing α subunits, the loss-of-function obtained through point mutation of one α subunit was smaller than the restriction-of-function in the respective triple point-mutated mouse. The discrepancies were particularly large for α3 in mechanical sensitization and for α5 in heat hyperalgesia, suggesting that these effects occur mainly through mixed GABA_A_Rs. Importantly, the rank orders of antihyperalgesic efficacy were the same for the restriction-of-function and loss-of-function approaches with α2>α5>α3 for mechanical sensitization and α2>α3>α5 for heat hyperalgesia and chemical nociception. The same rank order of efficacies has also been reported previously for antihyperalgesia using local spinal injections in single GABA_A_R point-mutated mice^5^. Our present results therefore corroborate the critical importance of α2GABA_A_Rs as targets for antihyperalgesia.

Our experiments on the quadruple point-mutated mice indicate that antihyperalgesia, anxiolysis, muscle relaxation and impairment of motor coordination occur through the high-affinity BDZ-binding site formed by the α/γ interface. Among the DZP actions assessed here, only sedation by high doses of DZP occurred in quadruple point-mutated mice. This result is consistent with previous electrophysiological data, which suggested the presence of a low-affinity BDZ-binding site in α1GABA_A_Rs contributing to the anaesthetic actions of BDZs^22^.

Most BDZ effects quickly diminish during prolonged treatment (that is, they undergo fast tolerance development). In the present study, we show that this is also the case for antihyperalgesia. Previous studies with the non-sedative BDZ site ligands L-838,417 and HZ-166 showed that these compounds have strongly reduced liabilities to tolerance development^5^,^20^,^27^. It has, however, not been possible to attribute this reduced tolerance to generally reduced agonistic activity or to improved subtype specificity. Our present study shows that tolerance can be avoided when only α2GABA_A_Rs are targeted, even with compounds that exert full agonistic activity. Additional experiments in RHRH and RHHR mice exclude α5GABA_A_Rs and suggest that activity at α3GABA_A_Rs is required for tolerance induction. However, because all these experiments were carried out in mice carrying the H→R point mutation in the α1 subunit, we cannot exclude that tolerance occurs through mixed α1/α3GABA_A_Rs, which in the wt situation may exhibit an α1 pharmacology. Such a scenario would explain why compounds with activity at α3GABA_A_Rs but absent or reduced activity at α1GABA_A_Rs lack liability to tolerance^5^,^20^,^27^. Interestingly, tolerance against the anxiolytic action of DZP was retained in triple point-mutated RHRR mice with only α2GABA_A_Rs remaining BDZ-sensitive. This difference suggests that tolerance development against the different BDZ effects involves activity at distinct subunits and possibly different mechanisms. Cell- or receptor-autonomous processes, which have been proposed for the desensitization of hippocampal α2GABA_A_Rs (ref. 25), are unlikely to be responsible for the tolerance against antihyperalgesia.

Addictive (reinforcing) properties of classical BDZs are another area of concern that has not been addressed in the present study. Reduced BDZ-induced reward facilitation has been reported in mice carrying the H→R point mutation in either the α1, α2 or α3GABA_A_R subunits^35^,^36^. Preference for MDZ in a two-bottle choice paradigm was absent in α1 (refs 31, 35) and α2 point-mutated mice^35^, suggesting that the simultaneous positive modulations of both the α1 and α2GABA_A_R are required for reinforcement. Tan *et al*.^32^ reported that α1GABA_A_Rs on GABAergic neurons in the ventral tegmental area (VTA) are required for MDZ-induced neuronal plasticity, strengthening glutamatergic excitation in the VTA. These findings suggest that activity at more than one GABA_A_R subtype is necessary for BDZ reinforcement. α2-selective BDZ site agonists might therefore be largely devoid of addictive properties.

Previous studies have provided evidence that activation of supraspinal GABA_A_Rs might enhance pain and counteract spinal antihyperalgesia, for example, through the inhibition of descending antinociceptive fibre tracts^37^,^38^,^39^. In our study, we did not observe any pronociceptive actions of systemically administered DZP. If such pronociceptive actions were relevant in the pain models used here, they would occur through α1GABA_A_Rs whose functions in nociception were not addressed in the present study. Any such effects would be avoided by α1-sparing BDZ site agonists.

The availability of the triple GABA_A_R point-mutated mice permitted a direct pharmacokinetic/pharmacodynamic comparison of α1-mediated sedation and α2-mediated antihyperalgesia in the absence of confounding behavioural effects from other GABA_A_R subtypes. In case of DZP, ED_50_ values for sedation and antihyperalgesia differed by a factor of more than 20, and a 50% higher degree of receptor occupancy was needed for α2-mediated antihyperalgesia compared with α1-mediated sedation. New subtype-selective agents will therefore have to have a high degree of α2 over α1 selectivity in order to achieve clinically relevant antihyperalgesia in the absence of sedation. These data also indicate that dose-limiting sedation most likely underlies the lack of clinically relevant antihyperalgesic effects of presently used (nonselective) BDZs. This is consistent with a recent clinical study in human volunteers showing weak antihyperalgesic effects at doses that caused only mild sedation^40^.

Which GABA_A_R subtype should be targeted for an optimal benefit-risk ratio in pain treatment? Both the present restriction-of-function and loss-of-function experiments on systemic BDZ administration and previous experiments with local spinal injections and single GABA_A_R point-mutated mice^5^ attribute the highest antihyperalgesic efficacy to α2GABA_A_Rs. The present study has shown that adding activity at α3GABA_A_Rs or α5GABA_A_Rs increases antihyperalgesic efficacy only moderately or not at all. Because it is at present not known how the different mixed GABA_A_Rs respond to subtype-selective agents, a conservative prediction should be made on the basis of the results obtained from triple point-mutated mice. These experiments indicate that sedation is exclusively due to activation α1GABA_A_Rs, which is also consistent with studies using the subtype-selective agonist TPA023B that completely lacks agonistic activity at α1GABA_A_Rs as well as sedation in humans^41^. The present experiments also showed that impaired motor coordination involves α1GAB_A_Rs and/or α3GABA_A_Rs. None of the undesired effects investigated in the present study could be attributed to α5GABA_A_Rs. However, cognitive impairment by nonselective BDZ likely originates from activity at α5GABA_A_Rs, as α5GABA_A_R-selective inverse agonists enhance cognitive capabilities^42^. Subtype-selective BDZ agonists targeting only α2GABA_A_Rs should therefore have the best benefit-risk ratio. They will produce pronounced antihyperalgesia in the absence of sedation and will not interfere with motor coordination and should not lose antihyperalgesic activity during prolonged treatment. They will however have anxiolytic properties (at least during the beginning of the treatment) and muscle-relaxant effects. Both of these actions may be beneficial in chronic pain patients. Because the side effects studied here are relevant for other indications, we expect that our present findings will benefit not only the development of innovative subtype-selective BDZs as analgesics but also as drugs for the treatment of several prevalent psychiatric diseases^43^.

## Methods

### Mice

Experiments were performed in wt mice, and in homozygous single, double, triple and quadruple (H→R) GABA_A_R point-mutated mice, that is, in mice expressing different combinations of BDZ-sensitive and BDZ-insensitive GABA_A_R α subunits. All mice were of the same genetic background (129X1/SvJ). Double, triple and quadruple point-mutated mice were generated by cross-breeding single point-mutated mice described earlier^11^,^12^,^44^.

### Autoradiography

The distribution of BDZ-sensitive GABA_A_R subtypes in the lumbar spinal cord was examined in 16-μm-thick horizontal sections, which were cut from fresh-frozen spinal cords. Sections were incubated with 5 nM [^3^H]flumazenil (50 Ci mmol^−1^) or 9 nM [^3^H]Ro15-4513 (22.7 Ci mmol^−1^) diluted in 50 mM Tris pH 7.4 for 120 min on ice. After washing three times for 20 s in ice-cold buffer, sections were dried and exposed along with [^3^H]-standards to a tritium-sensitive phosphoimaging screen (Cyclone Storage Phosphor Screen, Perkin Elmer). Quantification was carried out using the Optiquant software (Perkin Elmer). Nonspecific binding was assessed by co-incubating 10 μM clonazepam ([^3^H]flumazenil binding) or 10 μM flumazenil ([^3^H]Ro15–4513-binding).

### Immunohistochemistry

The localization of GABA_A_R subunits was visualized on 40-μm-thick lumbar spinal cord cryosections by DAB immunoperoxidase staining on sections from artificial cerebrospinal fluid (aCSF)-perfused mice postfixed for 90 min in 4% paraformaldehyde (PFA) (without picric acid)^45^. Antibodies were home-made subunit-specific antisera^45^. Final dilutions were 1:20,000 (α1), 1:1,000 (α2), 1:10,000 (α3), 1:3,000 (α5) and 1:10,000 (γ2). Images were taken with a bright field light microscope connected to a digital camera and processed with the AxioVision Rel. 4.5 software where an intensity-gradient false-colour filter was applied.

### Behavioural experiments

All behavioural experiments were performed in 7- to 12-week-old female and male mice. Care was taken to ensure equal numbers of female and male mice. All behavioural experiments were made by a male experimenter, blinded either to the genotype of the mice or to their treatment with drug or vehicle. Permission for animal experiments was obtained from the Veterinäramt des Kantons Zürich (licence numbers 135/2009 and 126/2012).

DZP (suspended in 0.9% saline and 1% Tween80) and MDZ (dissolved in 0.9% saline pH 3.0) were applied orally in all experiments. HZ-166 (suspended in 0.5% methyl cellulose) was given intraperitoneally (i.p.).

Neuropathic pain was evoked through CCI^17^ of the left sciatic nerve proximal to the trifurcation with three loose (5-0 silk) ligatures. Mice, which showed signs of paralysis or which did not develop significant hypersensitivity, were excluded from subsequent experiments. Effects of DZP and MDZ (p.o. in 0.9% saline and 1% Tween 80) on thermal and mechanical hyperalgesia were assessed 1 week after surgery. Heat hyperalgesia was quantified as the change in the latency of paw withdrawal evoked by exposure of the plantar side of one hindpaw to a defined radiant heat stimulus. Mechanical hyperalgesia was assessed with electronic von Frey filament no. 7 (IITC, Woodland Hills, CA). Three to four measurements were made for each time point and animal for both heat and mechanical hyperalgesia. Percent maximal possible effect (%MPE) was calculated as follows:

%MPE(*t*)=(*E*(*t*)−*E*_predrug_))/(*E*_preCCI_−*E*_predrug_); *E*(*t*), paw withdrawal thresholds or latency at time point *t*. *E*_predrug_, *E* after CCI surgery but before DZP application; *E*_preCCI_, *E* baseline before CCI surgery.

Formalin (4%, 20 μl) was injected subcutaneously into the plantar surface of the left hindpaw. Flinches and licks of the injected paw were counted for 1 h in 5-min intervals starting immediately after formalin injection. DZP was administered p.o. 60 min before formalin.

Locomotor activity was measured in an open field arena (10 cm diameter) equipped with four pairs of light beams and photosensors. Mice were placed into the arena 75 min before DZP application. Locomotor activity was analysed for the time interval between 20 and 165 min after DZP administration.

Motor control was assessed with a rotarod accelerating from 4 to 40 r.p.m. within 5 min. Sixty minutes after DZP administration mice were placed on the rotarod. Five measurements were taken per mouse.

To assess muscle relaxation, mice were placed with their forepaws on a metal horizontal wire placed 20 cm above ground. Successes and failures to grab the wire with at least one hindpaw were recorded between 60 and 180 min after DZP administration.

### Electrophysiology

The effects of DZP and MDZ on currents through recombinant GABA_A_Rs were studied in HEK293 cells (ATCC) transiently expressing GABA_A_Rs. HEK293 cells were transfected using lipofectamine LTX^46^. To ensure expression of the γ2 subunit (required for modulation of GABA_A_Rs by BDZs) in all recorded cells, we transfected cells with a plasmid expressing the γ2 subunit plus enhanced green fluorescent protein from an internal ribosomal entry site (IRES), and only selected eGFP-positive cells for recordings. The transfection mixture contained (in μg): 1 α1, 1 β2 and 3 γ2/eGFP (used as a marker of successful transfection) or 1 α2, 1 β3 and 3 γ2/eGFP. Recordings were made at 18–36 h after transfection. Whole-cell patch-clamp recordings of GABA-evoked currents were made at room temperature (20–24 °C) and at a holding potential of −60 mV. Recording electrodes were filled with solution containing (in mM): 120 CsCl, 10 EGTA, 10 HEPES (pH 7.40), 4 MgCl_2_, 0.5 GTP and 2 ATP. The external solution contained (in mM): 150 NaCl, 10 KCl, 2.0 CaCl_2_, 1.0 MgCl_2_, 10 HEPES (pH 7.4) and 10 glucose. GABA was applied to the recorded cell using a manually controlled pulse (4–6 s) of a low subsaturating GABA concentration (EC_5_). EC_5_ values of GABA were determined for wt α1β2γ2, wt α2β3γ2 and mutant α1(H101R)β2γ2 receptors. EC_50_ values and Hill coefficients (*n*_H_) were obtained from fits of normalized concentration–response curves to the equation *I*_GABA_=*I*_max_ [GABA]*n*_H_/([GABA]*n*_H_+[EC_50_]*n*_H_). *I*_max_ was determined as the average maximal current elicited by a concentration of 1 mM GABA. DZP and MDZ dissolved in DMSO and subsequently diluted with recording solution were co-applied together with GABA without preincubation.

### [^3^H]Ro 15-4513-binding assay to recombinant GABA_A_Rs

HEK293 cells were maintained in DMEM supplemented with 10% fetal bovine serum and seeded to a density of 500,000 cells on poly-lysine-coated 100-mm culture dishes 1 day before transfection. Cells were transfected with plasmids containing the subunit combination α1β2γ2 or α1(H101R)β2γ2 (7 mg total DNA, ratio 1:1:2) using jetPEI transfection reagent (Polyplus transfection). Twenty-four hours after transfection, HEK293 cells were harvested in PBS. HEK293 cells were homogenized in 10 mM Tris pH 7.5, protease inhibitor cocktail (complete Mini, Roche Applied Science). Aliquots of the homogenate prepared from HEK293 cells expressing the α1β2γ2 or α1(H101R)β2γ2 subunit combination were incubated with increasing concentrations of MDZ and 6.3 nM [^3^H]Ro15-4513 (22.7 Ci mmol^−1^, PerkinElmer) in a total volume of 200 μl for 90 min on ice. Subsequently, the samples were filtered on glass fibre filters using a 12-channnel semiautomated cell harvester (Scatron) and washed with ice-cold buffer (10 mM Tris-HCl pH 7.4). Nonspecific [^3^H]Ro15-4513 binding was measured using 10 μM flumazenil. The radioactivity of the filters was determined by liquid scintillation counting using a Tricarb 2500 liquid scintillation analyser.

### Receptor occupancy

Mice were killed immediately after completion of the behavioural tests, that is, 90 min after DZP administration and 60 min after MDZ administration. Brains and spinal cords were removed and kept frozen until further processing. Percentage of receptors occupied by DZP or MDZ was determined *ex vivo* with [^3^H]flumazenil as radioligand^47^. Frozen brains or lumbar spinal cords were rapidly homogenized in 20 volumes 10 mM Tris pH 7.4, 100 mM KCl and 100 μl aliquots were immediately added to 400 μl ice-cold buffer containing 4 nM [^3^H]flumazenil. After 30 s of incubation, samples were filtered on Whatman GF/C glass fibre filters and washed three times with 4 ml ice-cold buffer. Radioactivity retained on the filters was determined by scintillation counting. To determine nonspecific [^3^H]flumazenil binding, mice were used that had been injected i.p. with 5 mg kg^−1^ bretazenil (dissolved in PEG300) to fully occupy all BDZ-binding sites (100% receptor occupancy^47^).

## Additional information

**How to cite this article:** Ralvenius, W. T. *et al*. Analgesia and unwanted benzodiazepine effects in point-mutated mice expressing only one benzodiazepine-sensitive GABA_A_ receptor subtype. *Nat. Commun.* 6:6803 doi: 10.1038/ncomms7803 (2015).

## Figures and Tables

**Table 1:** Baseline nociceptive sensitivities of all GABA_A_R genotypes under study.

**Genotype**	**BDZ-sensitive α subunit (-s)**	**Pre-CCI**		**Post-CCI**	
		**von Frey (g) (number of mice)**	**Hargreaves (s) (number of mice)**	**von Frey (g) (number of mice)**	**Hargreaves (s) (number of mice)**
HHHH (wt)	α1, α2, α3 and α5	4.04±0.07 (20)	22.9±0.72 (17)	1.48±0.23 (8)	11.7±1.03 (16)
RHHH	α2, α3 and α5	4.41±0.11 (23)	22.6±0.63 (15)	1.52±0.11 (15)	9.68±0.81 (12)
RHHR	α2 and α3	4.21±0.12 (26)	23.4±0.57 (15)	1.46±0.09 (20)	10.4±1.15 (10)
RHRH	α2 and α5	4.05±0.09 (23)	22.9±0.60 (20)	1.26±0.10 (9)	12.8±1.70 (7)
RRHH	α3 and α5	4.23±0.04 (29)	21.0±0.43 (29)	1.52±0.06 (8)	8.54±0.62 (19)
RHRR	α2	4.12±0.06 (32)	22.2±0.53 (32)	1.42±0.11 (18)	10.7±0.72 (19)
RRHR	α3	4.12±0.12 (29)	22.2±0.50 (29)	1.27±0.08 (13)	10.3±1.00 (9)
RRRH	α5	4.32±0.07 (23)	21.5±0.42 (23)	1.67±0.08 (11)	10.2±0.89 (12)
RRRR	None	3.99±0.10 (9)	20.8±1.48 (6)	1.18±0.16 (9)	10.3±1.51 (6)
Statistics		F(8,205)=1.57; *P*⩾0.06	F(8,177)=1.93; *P*⩾0.06	F(8,102)=1.78; *P*⩾0.16	F(8,101)=1.80; *P*⩾0.09

ANOVA, analysis of variance; BDZ, benzodiazepine; CCI, chronic constriction injury; GABA_A_R, GABA_A_ receptor.

Values are given as mean±s.e.m.

ANOVA followed by Dunnett's *post hoc* test with wt (HHHH) as control.

**Table 2:** Baseline locomotor activity and performance in the horizontal wire and rotarod tests.

**Genotype**	**BDZ-sensitive α subunit (-s)**	**Activity counts (number of mice)**	Horizontal wire performance* **(number of mice)**	**Rotarod performance**† **(number of mice)**
HHHH (wt)	α1, α2, α3 and α5	962±114 (14)	92±2.5 (17)	92±5.6 (5)
HRRR	α1	1102±104 (9)	98±1.2 (9)	98±8.2 (6)
RHRR	α2	1132±106 (8)	96±1.2 (8)	81±5.3 (5)
RRHR	α3	1279±114 (10)	99±1.1 (10)	85±7.7 (5)
RRRH	α5	987±90.4 (8)	100±0.0 (8)	84±11 (5)
RRRR	None	1037±115 (9)	99±1.2 (9)	110±13 (5)
Statistics‡		F(5,52)=1.16; *P*⩾0.13	F(5,55)=2.10; *P*⩾0.70	F(5,25)=1.53; *P*⩾0.47

ANOVA, analysis of variance; BDZ, benzodiazepine; wt, wild type.

Values are given as mean±s.e.m.

^*^Horizontal wire performance, expressed as success rate (%).

^†^Rotarod performance, expressed as time before fall off (s).

^‡^ANOVA followed by Dunnett's *post hoc* test with wt (HHHH) as control.

**Table 3:** PK/PD parameters* of DZP and MDZ.

	**DZP**		**MDZ**	
	**α1/β2/γ2**	**α2/β3/γ2**	**α1/β2/γ2**	**α2/β3/γ2**
EC_50_ (μM)	0.081±0.02	0.15±0.04	0.17±0.18	0.092±0.019
*E*_max_ (% pot *I*_GABA_)	91.6±7.7	103±11	235±43	75.8±4.7
*n*_H_	1.50±0.60	1.20±0.35	0.73±0.26	1.23±0.29
α2/α1 selectivity†	1.25		0.32	
	Sedation	Analgesia	Sedation	Analgesia
ED_50_ (mg kg^−1^)	0.33±0.05	7.6±0.6	0.52±0.11	10.4±2.0
*E*_max_ (%MPE)	93.6±4.5	100 (Fixed)	92.2±10.4	100 (Fixed)
*n*_H_	2.0±0.64	0.75±0.19	2.28±0.98	1.42±0.40
ED_50_ (sedation/analgesia)	0.043		0.050	
	Sedation	Analgesia	Sedation	Analgesia
RO_50_ (%)	46.9±5.5	70.9±1.7	23.7±2.2	76.5±1.8
*E*_max_	97.9±7.1	100 (Fixed)	94.7±11.8	100 (Fixed)
Rate	14.1±4.1	14.9±2.0	4.14±2.10	5.01±2.11
RO_50_ (sedation/analgesia)	0.66		0.31	

PK, pharmacokinetic; PD, pharmacodynamic; DZP, diazepam; MDZ, midazolam; %MPE, percent maximal possible effect; RO, receptor occupancy.

For number of mice per group see Fig. 6.

^*^Values are mean±s.d.

^†^*E*_max_ (α2/β3/γ2)/*E*_max_ (α1/β2/γ2).
